# Microbe Profile: Streptomyces formicae KY5: an ANT-ibiotic factory

**DOI:** 10.1099/mic.0.001623

**Published:** 2025-10-24

**Authors:** Rebecca Devine, Katie Noble, Barrie Wilkinson, Matt Hutchings

**Affiliations:** 1 Department of Molecular Microbiology, John Innes Centre, Norwich Research Park, Norwich NR4 7UH, UK; 2 Centre for Microbial Interactions, Norwich Research Park, Norwich NR4 7UG, UK

**Keywords:** Actinomycetes, natural products, plant-ant mutualism, *Streptomyces*

## Abstract

*Streptomyces formicae* KY5 was isolated from a *Tetraponera penzigi* plant-ant nest. It is primarily known for its production of the formicamycins, antibiotics with potent activity against Gram-positive pathogens including methicillin-resistant *Staphylococcus aureus*, and additionally produces an antifungal compound that inhibits multi-drug-resistant fungal pathogens including *Lomentospora prolificans. S. formicae* is genetically tractable using CRISPR-Cas9 gene editing, allowing for detailed analysis of the formicamycin biosynthetic gene cluster. AntiSMASH analysis predicts the genome to encode at least 45 secondary metabolite biosynthetic gene clusters, many of which appear to encode novel compounds. Current research efforts are focussing on characterising the regulation of secondary metabolism at a global level in order to switch on pathways that are not typically expressed under standard laboratory conditions with the aim of identifying novel antimicrobials.

## Graphical Abstract

*Streptomyces formicae* was isolated from *Tetraponera penzigi* plant-ants and has bioactivity against Gram-positive pathogens due to the production of formicamycins.

## Taxonomy

Phylum, Actinomycetota (formerly known as Actinobacteria); class, *Actinomycetia*; order, *Streptomycetales*; family, *Streptomycetaceae*; genus, *Streptomyces*; species, *Streptomyces formicae*. The species name ‘*formicae*’ reflects its initial isolation from ants (*formica* being Latin for ant) [1].

## Properties

*S. formicae* is an aerobic, Gram-positive bacterium, which, like many other species of actinomycetes, begins life as a spore. These spores germinate into vegetative hyphae which grow into a dense mat of mycelium. In response to environmental signals, aerial hyphae are formed which eventually septate into chains of uni-genomic spores, ready for the life cycle to start again [2]. The mycelium of *S. formicae* is yellow in colour, due to the production of formicamycins. Spores are white and appear ‘fluffy’ much like other members of the genus. On rich solid media, *S. formicae* can complete its life cycle in 7–10 days. *S. formicae* can also grow in liquid culture; however, it does not sporulate and it does not produce the formicamycins.

## Genome

The genome of *S. formicae* was sequenced using PacBio and 454 platforms and reported in 2018. This showed it has a single linear chromosome of 9.6 Mbps in size, with a 71.4 G+C mol% [3].

## Phylogeny and ecology

*S. formicae* is one of ~1,100 verified *Streptomyces* species in the family Streptomycetaceae, order Streptomycetales, class Actinomycetes and phylum Actinomycetota. It was isolated from *Tetraponera penzigi* plant ants where they live in mutualism with their host plant the acacia tree *Vachellia drepanolobium* and cultivate a fungus in central Africa [4]. The trees have evolved specialised hollow structure called domatia that house and protect the ants and their food-fungus, and in return, the ants protect the plant from other herbivores through behavioural displays [5]. Analysis of the bacterial communities associated with these plant-ant symbioses revealed the presence of multiple species of antibiotic-producing bacteria, including Actinomycetes and Proteobacteria [6]. This type of mutualistic relationship of fungus-farming ants is well established; in particular, leafcutter ants are known to cultivate the *Leucoagaricus* fungus within their nests which provides their food source. These leafcutter ants carry a protective microbiome of actinomycete bacteria on their cuticle which produce antimicrobial compounds to protect the food-fungus from disease and other microbes [7,9]. The *T. penzigi* ants may show another example of this, with *S. formicae* and its natural products contributing to the protective mutualism of their relationship.

## Key features and discoveries

*S. formicae* was initially identified as a strain of interest due to its broad-ranging bioactivity. Under standard laboratory conditions, *S. formicae* produces molecules that inhibit both Gram-positive bacteria and fungi, including the drug-resistant pathogens methicillin-resistant *Staphylococcus aureus* and *Lomentaspora prolificans*. AntiSMASH analysis of the *S. formicae* genome shows that it encodes at least 45 biosynthetic gene clusters (BGCs), which is considerably more than average for a strain of *Streptomyces* [1,3]. Furthermore, most of these BGCs have the potential to produce novel compounds, suggesting that mining this strain may result in the discovery of several new molecules.

The antibacterial activity of *S. formicae* is due to the production of the formicamycins, a novel family of aromatic polyketide antibiotics. Formicamycins are related to fasamycins, which were initially identified from the heterologous expression of environmental DNA isolated from soil samples collected from Arizona [10]. The antifungal activity of *S. formicae* is due to the production of a so-far uncharacterized molecule. *S. formicae* is genetically tractable using CRISPR-Cas9, allowing researchers to fully delineate the steps of formicamycin biosynthesis and regulation, and genetic rewiring has resulted in overproduction of multiple pathway products, biosynthetic intermediates and shunt metabolites [11,13]. Current research efforts are using gene editing techniques to rewire the regulation of secondary metabolism in *S. formicae* in order to switch on silent pathways and discover novel molecules. This work shows how exploring secondary metabolism in new strains can reveal insights into biosynthetic pathways as well as allow for the discovery of new bioactive molecules.

## Open questions

- Why are the other BGCs not expressed in the wild-type strain?
- Do any of these other BGCs encode novel antimicrobials?
- Which regulators control secondary metabolism in *S. formicae* on a global level?
- Do plant-ant systems harbour other novel antibiotic-producing species?

## Abbreviations

BGC: biosynthetic gene cluster
CRISPR: clustered regularly interspaced short palindromic repeats
